# Bevacizumab for ramucirumab refractory malignant pleural effusion in non-small cell lung cancer: a case report and review of the literature

**DOI:** 10.18632/oncotarget.17952

**Published:** 2017-05-17

**Authors:** Ryobu Mori, Daichi Fujimoto, Munehiro Ito, Keisuke Tomii

**Affiliations:** ^1^ Department of Respiratory Medicine, Kobe City Medical Center General Hospital, Hyogo, Japan

**Keywords:** bevacizumab, malignant pleural effusion, non-small cell lung cancer, ramucirumab, vascular endothelial growth factor

## Abstract

Malignant pleural effusion (MPE) is a major problem associated with advanced non-small cell lung cancer for which an optimum treatment strategy has yet to be determined. Notably, vascular endothelial growth factor (VEGF) signaling has been found to influence MPE, and bevacizumab, a VEGF ligand inhibitor, can effectively control MPE. Ramucirumab, a human monoclonal antibody specific for VEGF receptor-2, has recently been approved for advanced non-small cell lung cancer. However, it remains unclear which of these agents more effectively control MPE.

We describe a case of a 68-year-old man with advanced non-small cell lung cancer in whom ramucirumab plus docetaxel-refractory MPE was responsive to bevacizumab plus docetaxel combination therapy. The patient’s MPE progressed after two cycles of ramucirumab plus docetaxel second-line chemotherapy. After switching to bevacizumab plus docetaxel, a computed tomography scan revealed a decreased MPE after two cycles of treatment.

Bevacizumab may be more effective for treating MPE. However, further investigations are still warranted to determine the optimal VEGF-targeted agent for this condition.

## INTRODUCTION

Non-small cell lung cancer (NSCLC) accounts for approximately 80% of lung cancer cases, the majority of which are already unresectable and metastatic at the time of initial diagnosis. Additionally, approximately 40% of patients with advanced NSCLC will present with or develop malignant pleural effusion (MPE) [1]. Pleurodesis is considered a standard procedure for treating MPE. However, this treatment has no effect on the tumor itself and only provides symptomatic relief. Chemical pleurodesis is associated with adverse events, such as fever, chest pain, and acute respiratory distress syndrome. Additionally, the process of intercostal tube insertion itself can cause complications, including hemorrhage, organ injury, and infection. Consequently, optimum strategies for managing recurrent effusions are currently unavailable [2].

Vascular endothelial growth factor (VEGF) is thought to play a principal role in MPE formation by increasing vascular and mesothelial permeability and capillary fluid leakage [3]. As bevacizumab, an anti-VEGF-A agent, was found to effectively control pleural effusion in clinical studies [4, 5], attention has focused on the use of VEGF axis inhibitors to treat patients with MPE. Ramucirumab, a human monoclonal antibody specific for VEGF receptor-2 (VEGFR-2), was recently approved for patients with advanced NSCLC [6]. Bevacizumab and ramucirumab mainly differ with respect to their drug target. Bevacizumab is an antagonist of the VEGF ligand, VEGF-A, whereas ramucirumab is an antagonist of VEGFR-2. However, it remains unclear which of these agents more effectively control MPE in a clinical setting. Herein, we report on a case of MPE that was refractory to ramucirumab plus docetaxel, but responsive to bevacizumab plus docetaxel combination therapy and review the literature.

## CASE PRESENTATION

A 68-year-old Japanese man presented at our hospital with dyspnea. A computed tomography (CT) scan revealed right-sided pleural effusion and a pulmonary nodule. Radiographic and pathologic evaluations confirmed a diagnosis of advanced pulmonary adenocarcinoma with MPE (T1aN0M1a, Stage 4). Epidermal growth factor receptor mutation analysis and anaplastic lymphoma kinase fusion gene testing were negative. Carboplatin, paclitaxel, and bevacizumab (15 mg/kg) was administered as first-line chemotherapy, and after 4 cycles, a CT scan indicated the disappearance of the right-sided pleural effusion and a partial tumor response. Bevacizumab maintenance therapy was discontinued due to patient preference.

**Figure 1:**
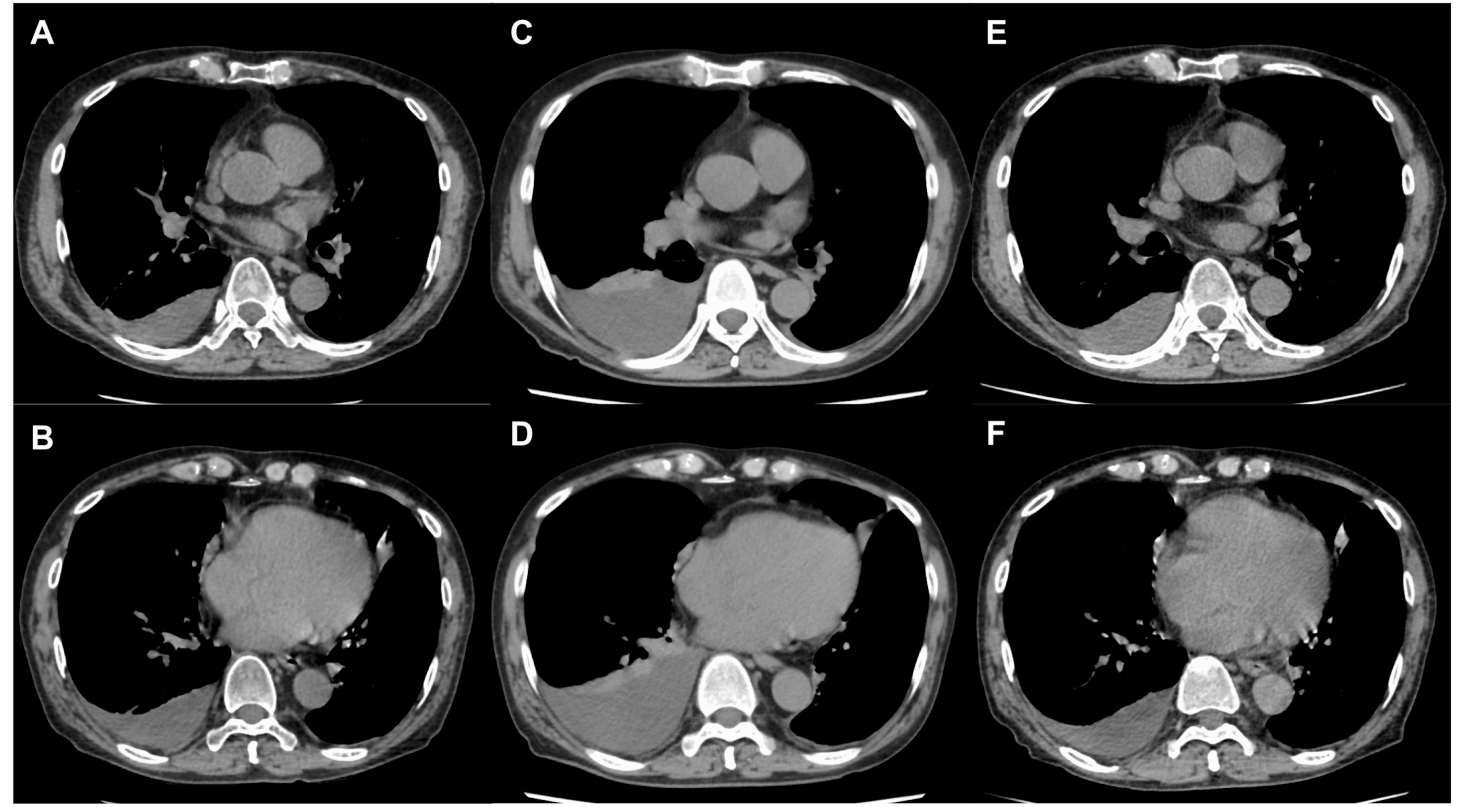
Changes in MPE with treatment MPE **A**., **B**. at initiation of ramucirumab plus docetaxel and **C**., **D**. after 2 cycles of ramucirumab plus docetaxel; MPE increased and progressive disease was confirmed. MPE decreased after 2 cycles of bevacizumab plus docetaxel **E**., **F**.

**Figure 2:**
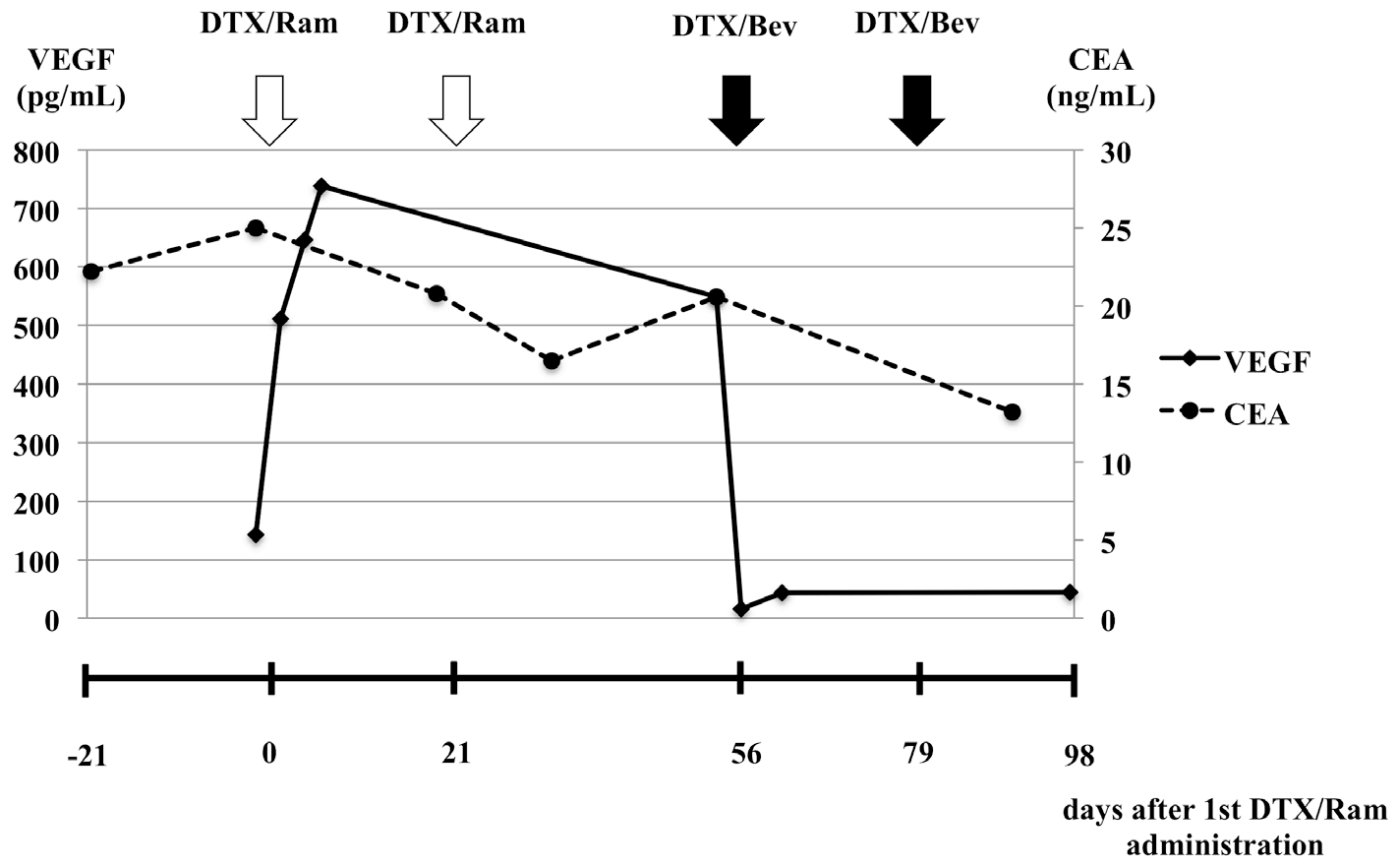
Changes in serum VEGF and carcinoembryonic antigen levels before and after ramucirumab plus docetaxel and bevacizumab plus docetaxel combination therapy

Three months after the final administration of first-line chemotherapy, progressive disease was detected as an increase in pleural effusion on a CT scan and pleural fluorodeoxyglucose uptake. Despite administering ramucirumab (10 mg/kg, Day 1) plus docetaxel (60 mg/m^2^, Day 1) as second-line chemotherapy, a CT scan revealed that, after 2 cycles, the pulmonary nodules were unchanged, but the pleural effusion had increased. Therefore, the MPE was considered progressive. Serum VEGF-A levels increased from 143.0 pg/mL before second-line chemotherapy to 511.0 pg/mL after second-line chemotherapy. This high level of serum VEGF-A was maintained after 2 treatment cycles.

Pleurodesis was not considered indispensable at this point, because the patient did not have severe symptoms of MPE after administration of ramucirumab plus docetaxel combination therapy. Subsequently, bevacizumab (15 mg/kg, Day 1) plus docetaxel (60 mg/m^2^, Day 1) was administered for progressive MPE. After 2 cycles, a CT scan revealed a reduction in the pleural effusion with the pulmonary nodule remaining stable. Moreover, the patient's serum VEGF-A levels had decreased from 549.0 pg/mL before administration to <15.6 pg/mL on Day 3 of the first treatment course. A response was maintained for 4 months after the administration of bevacizumab plus docetaxel combination therapy and has continued to be maintained.

## DISCUSSION

The family of VEGF ligands comprises VEGF-A, VEGF-B, VEGF-C, VEGF-D, VEGF-E, and placental growth factor. VEGF-A is important for vascular permeability and angiogenesis [7]. Its levels are significantly elevated in MPEs relative to pleural effusions associated with benign diseases [8]. Therefore, VEGF-A has been considered the most important VEGF ligand for MPE formation, and this has been supported by several clinical studies [4, 5] that have demonstrated the efficacy of bevacizumab, a VEGF-A inhibitor, for the treatment of MPE.

The family of VEGFRs includes VEGFR-1 (Flt-1), VEGFR-2 (KDR/Flk-1), and VEGFR-3 (Flt-4), among which VEGFR-2 was found to be essential for the induction of VEGF biological responses [7]. Notably, the REVEL trial [6] demonstrated that ramucirumab prolonged overall survival when administered in combination with docetaxel to patients with previously treated NSCLC. Although additional reports [9, 10] have demonstrated the importance of VEGFR-2 in MPE formation *in vitro*, the functions of the remaining VEGFRs have yet to be fully elucidated [7]. Thus far, it has been shown [11, 12] that VEGFR-1 is also upregulated in MPEs, and it is thought that VEGFR-1 may play a positive regulatory role in angiogenesis under certain conditions.

In the present case, bevacizumab plus docetaxel combination therapy was administered, although this is not considered a standard therapy. However, this therapy was proven to be associated with prolonged progression-free survival compared to docetaxel monotherapy in patients previously treated with bevacizumab-containing platinum-based doublet regimens [13]. Considering the findings of this trial [13], and previous efficacy data for bevacizumab in MPEs, we commenced treatment with this combination therapy in our patient.

In the present case, the MPE was refractory to ramucirumab plus docetaxel, but responsive to bevacizumab plus docetaxel combination therapy. Similar to previous reports [14, 15], our patient exhibited increased serum VEGF-A levels after ramucirumab therapy. We speculate that the inhibition of VEGFR-2 leads to elevated serum VEGF-A, which in turn binds to VEGFRs other than VEGFR-2. Increased MPE during ramucirumab therapy may be maintained via this mechanism. We believe that our report provides scope for improving treatment for MPEs, as well as, important clues to the mechanism(s) of association between VEGF and MPEs.

VEGF-A was not measured in the MPE in our case. Hsu *et al*. [16] examined serum and pleural effusion VEGF concentrations and reported a statistically significant correlation. Therefore, we believed serum VEGF would be a good surrogate for VEGF concentrations in the MPE in our case.

In conclusion, we demonstrate the efficacy of bevacizumab in a patient with ramucirumab refractory MPE. Although both agents inhibit the VEGF pathway, bevacizumab may be more effective for treating MPE. Further investigations are still warranted to determine the optimal VEGF-targeted agent for MPE.

## Abbreviations

CT: computed tomography
MPE: malignant pleural effusion
NSCLC: non-small cell lung cancer
VEGF: vascular endothelial growth factor
VEGFR: vascular endothelial growth factor receptor.
